# Adult degenerative scoliosis: challenges in diagnosis, pain management, and surgical decision-making

**DOI:** 10.3389/fsurg.2026.1807406

**Published:** 2026-07-07

**Authors:** Shuiwang Zhao, Jiaxin Liu, Bei Li, HaoLi Zhang, Rong Tian, Shenqiao Yang, Li Zhu

**Affiliations:** 1 Dazhou Vocational College of Chinese Medicine, Dazhou, China; 2 Rehabilitation Medicine Center/Tuina Department, Hubei Provincial Hospital of Traditional Chinese Medicine, Wuhan, China; 3 Hubei Shizhen Laboratory, Wuhan, China; 4 Affiliated Hospital of Hubei University of Chinese Medicine, Wuhan, China; 5 School of Acupuncture and Massage, Chengdu University of Traditional Chinese Medicine, Chengdu, China; 6 Acupuncture and Moxibustion Rehabilitation Center, Ankang Hospital of Traditional Chinese Medicine, Ankang, China; 7 Enshi Tujia and Miao Autonomous Prefecture Central Hospital, Enshi, China; 8 Rehabilitation College, Chengdu University of Traditional Chinese Medicine, Chengdu, China

**Keywords:** adult degenerative scoliosis, adult spinal deformity, complications, decompression, fusion, osteotomy, pain management, sagittal balance

## Abstract

**Background:**

Adult Degenerative Scoliosis (ADS) is an age-related, progressive, three-dimensional spinal deformity driven by asymmetric degeneration of the intervertebral discs, facet joints, and supporting soft tissues. With population aging, ADS is increasingly recognized as a major contributor to pain, disability, loss of independence, and healthcare utilization.

**Objective:**

This narrative review synthesizes evidence around three core challenges in the clinical management of ADS: (1) diagnostic difficulty due to heterogeneous etiology and symptom overlap with other degenerative conditions, (2) multidimensional chronic pain requiring multimodal, longitudinal strategies, and (3) high-stakes surgical decision-making that must balance deformity correction against complication risk and patient expectations.

**Methods and key content:**

We integrate contemporary concepts from adult spinal deformity classification and alignment targets, clinical and imaging assessment, and both nonoperative and operative treatment pathways. Particular emphasis is placed on standing full-spine radiographs and spinopelvic parameters, the mixed mechanical and neurogenic pain phenotypes that typify ADS, and stratified surgical strategies ranging from decompression alone to long-segment fusion with osteotomy. We also summarize major complications (e.g., proximal junctional kyphosis, pseudarthrosis, implant failure, infection) and their implications for counseling and risk mitigation.

**Conclusion:**

ADS care benefits from a patient-centered, individualized, and multidisciplinary strategy. Future work should prioritize accurate prognostic models, minimally invasive and alignment-restoring techniques with lower morbidity, and durable approaches for long-term pain control and function preservation.

## 1 Introduction

### 1.1 Definition and epidemiology

Adult Degenerative Scoliosis (ADS) refers to a coronal spinal curvature that develops or significantly progresses in adulthood as a consequence of degenerative changes in the motion segments of the spine. Although definitions vary across studies, ADS is commonly operationalized as a patient aged 18 years or older with a coronal Cobb angle greater than 10° on standing radiographs, accompanied by radiographic features of degeneration such as disc height loss, osteophytes, endplate sclerosis, and facet arthropathy (1). In contrast to adolescent idiopathic scoliosis, ADS is not merely a “curve problem”; it is a complex disorder of spinal aging in which deformity, pain, neurologic compromise, and systemic comorbidity interact (2). Epidemiologic estimates of ADS prevalence differ depending on cohort characteristics (community-dwelling vs referral-based), the threshold Cobb angle used, and whether “*de novo*” degenerative scoliosis is separated from progression of pre-existing idiopathic curves. In general, prevalence rises steeply with age, and ADS is increasingly encountered in routine spine practice in aging societies (3). Importantly, the burden of ADS is not limited to radiographic deformity; patients may experience reduced mobility, difficulty with standing or walking endurance, sleep disruption, emotional distress, and loss of social participation. These impairments drive health service use through primary care visits, imaging, pharmacologic therapy, injections, physical therapy, and ultimately surgery for selected patients.

From a health-system perspective, ADS occupies a challenging space: it is common, chronic, and heterogeneous, yet the most definitive intervention (major reconstructive surgery) is resource-intensive and associated with a non-trivial complication and revision burden. This context underlines why improved diagnostic precision, durable pain management, and transparent shared decision-making are essential.

### 1.2 Overview of pathophysiology

**Figure 1:**
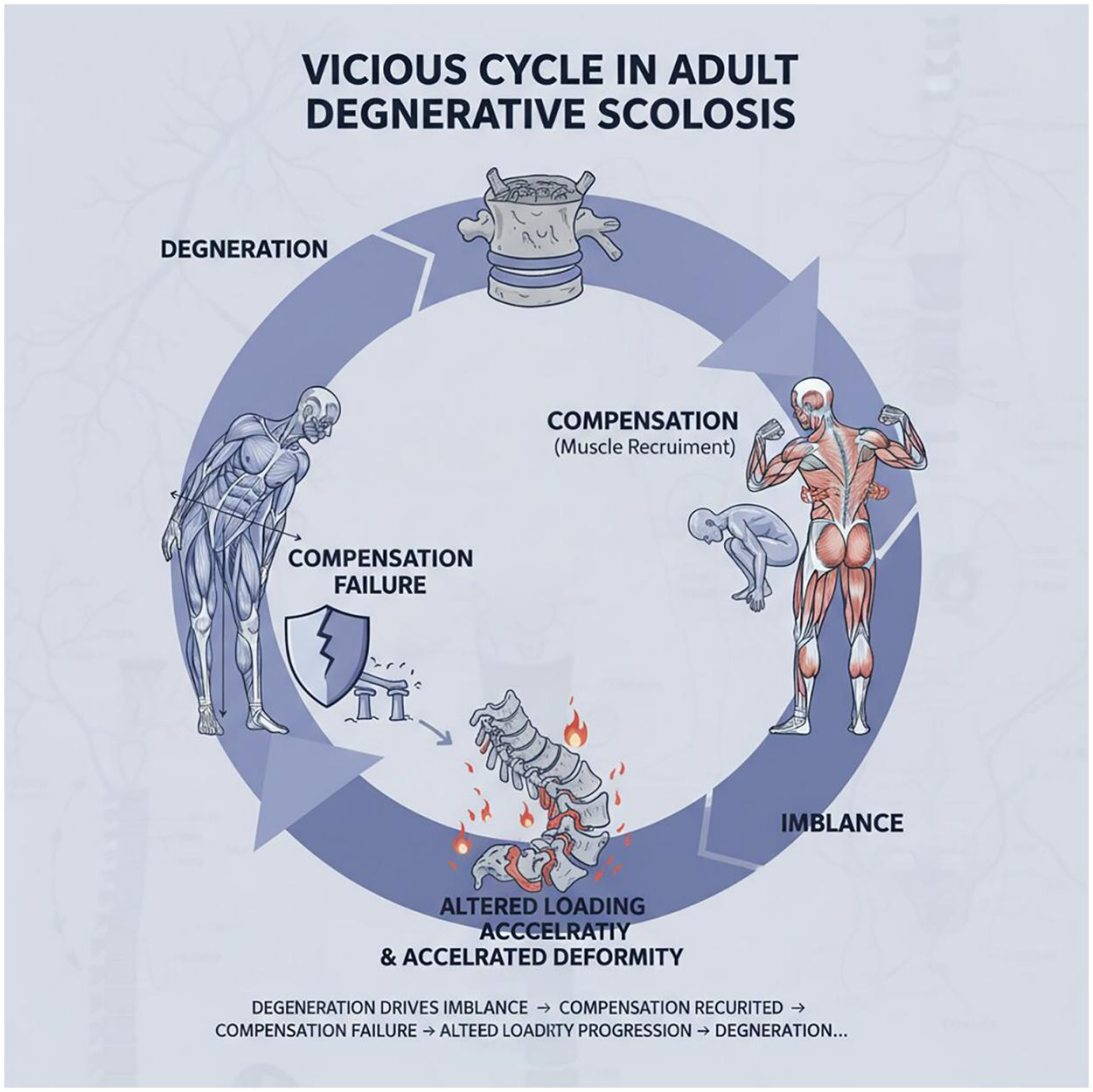
Vicious cycle in adult degenerative scoliosis (this figure was generated with the assistance of an AI language model (GLM-5) based on the author's conceptual framework. The author takes full responsibility for the accuracy and content of the figure).

ADS emerges from a multifactorial “degeneration–imbalance–compensation–progression” cycle. A graphical summary of the pathophysiological mechanism is presented in Figure 1. Asymmetric disc degeneration and facet joint arthrosis can create segmental instability and a tendency toward rotation and lateral translation. These changes alter load-sharing across the spine, which can accelerate further degeneration in a self-reinforcing manner. Paraspinal muscles, which contribute to dynamic stability and postural control, may undergo fatty infiltration and atrophy, further reducing the spine's ability to resist deforming forces.

Sagittal alignment deserves particular emphasis. Even modest coronal curves can coexist with clinically important sagittal plane malalignment. When lumbar lordosis diminishes due to disc collapse and degenerative kyphosis, the trunk tends to pitch forward. Patients may compensate through pelvic retroversion, hip extension, and knee flexion (4). These compensations are metabolically costly and often fail over time, leading to decompensated sagittal imbalance with substantial functional limitation (5). Contemporary adult spinal deformity frameworks highlight the strong correlation between sagittal malalignment and disability, and they emphasize alignment targets that are individualized to pelvic morphology (e.g., pelvic incidence) and age-related norms (6, 7).

Pathophysiology also includes neural element compromise. Hypertrophy of ligamentum flavum, osteophytes, disc bulging, and facet overgrowth can narrow the central canal, lateral recess, and foramen (8). In ADS, stenosis is often multilevel and asymmetric, resulting in neurogenic claudication and radiculopathy superimposed on mechanical back pain (9).

### 1.3 Presenting the core challenges

Compared with adolescent idiopathic scoliosis, ADS patients frequently present with multiple comorbidities (osteoporosis, sarcopenia, diabetes, cardiovascular disease), polypharmacy, and functional vulnerability (10). Consequently, clinical goals shift from maximal curve correction toward symptom relief, durable function, and maintenance of independence. Three challenges are consistently encountered.

First, diagnosis is often delayed or imprecise because symptoms overlap with other degenerative conditions such as lumbar spinal stenosis without deformity, degenerative spondylolisthesis, hip osteoarthritis, and vascular claudication. Second, pain in ADS is typically multifactorial, combining mechanical, neurogenic, and myofascial components that evolve over time; effective management requires multimodal strategies rather than a single “best” intervention. Third, surgical decision-making is complex because operations range from focal decompression to long-segment fusion with osteotomy, and complication risks increase with age, frailty, and deformity severity. This review addresses these challenges in sequence, focusing on clinically actionable concepts and patient-centered trade-offs.

## 2 The challenge of diagnosis: from symptom differentiation to precise assessment

### 2.1 Non-specific clinical presentation

ADS rarely declares itself with a pathognomonic symptom. Many patients present primarily with chronic low back pain, often described as aching, activity-related, and worse with prolonged standing (11). Others present with leg symptoms including radicular pain, paresthesia, weakness, or neurogenic claudication characterized by limited walking tolerance and symptom relief with sitting or forward flexion. These symptom patterns overlap substantially with lumbar spinal stenosis, degenerative spondylolisthesis, and discogenic pain syndromes. A further layer of complexity is that pain location does not reliably map to the deformity apex or the most degenerative segment. For example, a patient with a moderate lumbar curve may have dominant pain from a severely arthritic facet joint at an adjacent level, while another patient may have minimal back pain yet severe disability from multilevel foraminal stenosis. Additionally, coronal trunk shift and sagittal forward stooping can manifest as fatigue, decreased endurance, and balance problems rather than “pain” perse (12).

History should therefore explore multiple domains: onset and tempo of symptoms, activity limitations, neurologic complaints, red flags, prior spine interventions, and the presence of hip or vascular symptoms. Physical examination should include global posture assessment (coronal and sagittal), gait evaluation, hip range of motion, neurologic testing, and screening for myofascial tenderness and sacroiliac joint signs. Importantly, symptom severity is best understood in terms of function: sitting tolerance, standing time, walking distance, and ability to perform instrumental activities of daily living.

### 2.2 Complexity of imaging assessment

#### 2.2.1 Core role of full-spine radiographs

Standing full-spine anteroposterior and lateral radiographs remain foundational because they capture global alignment under physiologic load and allow standardized measurement of deformity and balance parameters. Coronal measures include Cobb angle, coronal imbalance (e.g., C7 plumb line offset), apical vertebral rotation, and lateral listhesis. Sagittal measures include sagittal vertical axis (SVA), pelvic tilt (PT), sacral slope (SS), pelvic incidence (PI), lumbar lordosis (LL), thoracic kyphosis, and the PI–LL mismatch, which is a widely used indicator of lordosis adequacy relative to pelvic morphology (6).

These parameters are not merely descriptive. They correlate with health-related quality of life measures and inform alignment targets for reconstruction (13–15). For instance, patients with marked positive sagittal balance may experience disabling energy expenditure during standing and walking. Conversely, a patient with a significant coronal curve but preserved sagittal alignment may function relatively well and may be managed nonoperatively or with limited surgery aimed at neural decompression.

A practical diagnostic pitfall is reliance on supine imaging alone. Supine radiographs or MRI can underestimate the true magnitude of coronal and sagittal imbalance because they remove the effect of gravity and muscular fatigue. Therefore, clinicians should prioritize upright imaging and, where available, low-dose biplanar radiography systems that reduce radiation while enabling three-dimensional analysis.

#### 2.2.2 Complementary roles of CT and MRI

While radiographs define alignment, cross-sectional imaging refines anatomic diagnosis and guides interventions. Computed tomography (CT) is valuable for evaluating bony anatomy, facet arthrosis, pars defects, vacuum phenomenon, and endplate sclerosis. It is also helpful in surgical planning for instrumentation trajectories in dysmorphic pedicles and for assessing prior fusion mass and hardware.

Magnetic resonance imaging (MRI) provides critical information on neural compression patterns and soft-tissue pathology. In ADS, stenosis may occur in the central canal due to ligamentum flavum hypertrophy and disc bulging; in the lateral recess due to facet overgrowth; or in the foramen due to disc height loss, vertebral rotation, and asymmetric collapse (8). MRI allows characterization of compression severity and laterality, identification of synovial cysts, and assessment of adjacent segment degeneration.

Notably, imaging findings frequently do not correlate perfectly with symptoms. Multilevel stenosis is common in older adults, and the most severe radiographic stenosis may be clinically silent (9). Therefore, imaging should be interpreted through the lens of symptom distribution, neurologic findings, and, when needed, selective diagnostic blocks.

#### 2.2.3 Value and limitations of dynamic imaging

Dynamic flexion–extension radiographs can help identify instability, including translational motion at degenerative spondylolisthesis levels and motion at segments with disc collapse and facet degeneration. In ADS, instability may also manifest as lateral listhesis that changes with posture. Recognizing instability matters because it influences the durability of decompression-alone strategies and may indicate the need for fusion.

However, dynamic radiographs have limitations. Patient pain and stiffness may reduce true range of motion, leading to underestimation of instability. In addition, micro instability and facet-mediated pain can exist without clear radiographic translation. Consequently, dynamic imaging should complement, not replace, clinical reasoning.

### 2.3 Key points in differential diagnosis

Differential diagnosis begins with distinguishing ADS from other scoliosis types. Some adults have progression of adolescent idiopathic scoliosis rather than *de novo* degenerative scoliosis. These entities may differ in curve patterns (e.g., thoracic involvement), rotational deformity, and compensation strategies, which can influence surgical planning (1).

Secondary scoliosis due to neuromuscular disorders, metabolic bone disease, vertebral fractures, infection, or tumors must be considered when history suggests atypical features (rapid progression, night pain, systemic symptoms, severe osteoporosis, or neurologic signs) (10). Additionally, non-spinal diseases can mimic ADS symptoms: hip osteoarthritis can present with groin pain and limited hip internal rotation; vascular claudication can mimic neurogenic claudication but is more related to exertion and less relieved by spinal flexion; and peripheral neuropathy can cause sensory changes unrelated to root distribution.

From a pragmatic standpoint, clinicians should ask two questions: (1) Is the patient symptomatic primarily from deformity-related imbalance, from stenosis-related neural compression, or from localized mechanical pain? (2) Are there non-spinal contributors to disability that would limit the benefit of spine-specific interventions? Answering these questions sets the stage for effective pain management and surgical decision-making.

## 3 The challenge of pain management: comprehensive strategies beyond a single modality

### 3.1 Multifactorial nature of pain sources

Pain in ADS is best conceptualized as a combination of overlapping generators.

**Mechanical low back pain** often arises from asymmetric loading, segmental instability, disc degeneration, and facet arthropathy. Patients may describe worsening pain with standing, extension, or prolonged activity and partial relief with rest or positional change (16).

**Neurogenic pain** includes radicular pain from foraminal stenosis and neurogenic claudication from central canal stenosis. Symptoms may be unilateral or bilateral and can be disproportionate to coronal curvature magnitude (9). Neurogenic pain commonly limits walking and may drive patients toward surgical consultation.

**Secondary myofascial pain** results from muscular fatigue and compensatory overuse. Paraspinal, gluteal, and hip flexor muscles may develop trigger points and tenderness, especially in patients with sagittal imbalance who rely on compensatory postures.

In many patients, these components fluctuate over time. A period of stable mechanical pain may be followed by abrupt onset of radicular symptoms due to foraminal collapse, or symptoms may shift after a fall or after progressive decompensation of sagittal alignment. Therefore, pain management in ADS should be framed as longitudinal, adaptive care that aligns short-term symptom control with long-term function preservation (17).

### 3.2 Non-surgical pain management strategies

#### 3.2.1 Physical therapy and rehabilitation

Rehabilitation is often first-line and remains important even for patients ultimately treated surgically. Core elements include trunk stabilization, hip and pelvic strengthening, flexibility work, aerobic conditioning, and posture education. The rationale is twofold: improving neuromuscular control may reduce mechanical pain and improve endurance, and enhancing general conditioning may mitigate frailty and improve surgical resilience if surgery becomes necessary (11).

Evidence for specific exercise protocols in ADS is evolving. Many studies are limited by heterogeneity and small sample sizes, yet clinical experience supports individualized programs that consider curve pattern, sagittal balance, pain phenotype, and comorbidities. For example, a patient with significant sagittal imbalance may benefit from endurance-based extensor strengthening and gait training, while a patient with dominant stenosis symptoms may prioritize cycling, aquatic therapy, and flexion-tolerant conditioning.

A key practical challenge is adherence. Older adults may face transportation barriers, fear of pain exacerbation, or competing medical appointments. Incorporating home-based components, setting measurable functional goals (e.g., increasing walking time), and coordinating with pain interventions can enhance engagement.

#### 3.2.2 Pharmacological treatment

Medication management in ADS must account for efficacy, side effects, and geriatric vulnerabilities (17). Acetaminophen may be used for baseline analgesia, though benefit for chronic spine pain is often modest. Non-steroidal anti-inflammatory drugs (NSAIDs) can reduce inflammatory pain but carry risks of gastrointestinal bleeding, renal dysfunction, and cardiovascular events, especially with long-term use in older adults.

Neuropathic pain medications such as gabapentin or pregabalin are commonly used for radicular symptoms. Potential benefits must be weighed against dizziness, sedation, cognitive effects, edema, and fall risk. Tricyclic antidepressants or serotonin-norepinephrine reuptake inhibitors may be considered in selected patients, particularly when sleep disruption or mood symptoms coexist.

Opioids should be used cautiously. While short courses may be appropriate for severe exacerbations, chronic opioid therapy is associated with tolerance, constipation, endocrine effects, cognitive impairment, and dependence, and it may not meaningfully improve function. In ADS, the ultimate goal of pharmacologic therapy should be to enable activity, sleep, and participation in rehabilitation rather than to eliminate pain entirely.

Polypharmacy is common. A structured medication review should evaluate drug–drug interactions, renal and hepatic function, and the presence of medications that increase fall risk. Shared decision-making and clear stop rules (e.g., trial periods with functional endpoints) can help avoid ineffective long-term regimens.

#### 3.2.3 Interventional procedures

Interventional pain procedures serve a dual diagnostic and therapeutic role in ADS management. Diagnostically, they help localize the primary pain generator (e.g., specific nerve root, facet joint) in the setting of multilevel pathology. Therapeutically, they can provide temporary symptom relief, facilitating participation in rehabilitation and improving quality of life, though their effects are often time-limited.

##### 3.2.3.1 Epidural steroid injections (ESIs)

Therapeutically, ESIs may provide short-term relief for neurogenic claudication or radicular pain. Diagnostically, a positive response can support the clinical suspicion of inflammation-mediated neural compression, though the level-specificity is lower than selective blocks.

##### 3.2.3.2 Selective nerve root blocks (SNRBs)

These serve a primarily diagnostic purpose to confirm the symptomatic level in multilevel foraminal stenosis. When followed by a corticosteroid, they offer therapeutic short-term radicular pain relief. A precise diagnostic block can crucially refine surgical planning.

##### 3.2.3.3 Facet joint interventions

Medial branch blocks are diagnostic to confirm facet-mediated mechanical pain. Radiofrequency ablation, when blocks are positive, provides therapeutic intermediate-duration (6–12 months) pain relief. However, their benefit is limited in the setting of gross instability (16).

##### 3.2.3.4 Sacroiliac joint injections

These are both diagnostic (to confirm SI joint pain, which mimics lumbar or gluteal pathology) and therapeutic (providing temporary relief, often with corticosteroid).

Interventions should be integrated into a broader plan rather than applied as repetitive standalone procedures. A useful framework is to define the intended function-enabled outcome (e.g., walk 20 min, complete a strengthening program, avoid escalation to opioids) and to reassess after each procedure whether that outcome has been achieved.

### 3.3 Link between pain management and quality of life

Pain intensity alone is an incomplete outcome metric in ADS. Many patients tolerate moderate pain if function is preserved, while others experience profound disability with similar pain scores due to imbalance, fear-avoidance, depression, or neurologic limitation (18).

Therefore, management should incorporate patient-reported outcomes and functional measures, such as walking distance, standing tolerance, and ability to perform valued activities. Shared goal setting can transform the clinical conversation from “fix my curve” to “help me walk to the store” or “help me stand long enough to cook”. This reframing is particularly important when discussing surgery, where alignment correction may improve function but cannot guarantee pain elimination.

Psychological factors matter. Chronic pain is associated with catastrophizing, anxiety, and sleep disturbance, which can amplify perceived disability (19). Screening and, when indicated, referral for cognitive-behavioral strategies, sleep optimization, and mood management can improve outcomes and may reduce reliance on procedures or medications.

In summary, pain management in ADS is best delivered as a coordinated program that combines education, graded activity, judicious medications, and targeted interventions, continuously aligned with functional goals and evolving pathology.

## 4 The challenge of surgical decision-making: balancing risks, benefits, and patient expectations

### 4.1 Consensus and controversy in surgical indications

Surgery is generally considered when nonoperative management fails to provide acceptable function or when neurologic compromise progresses. Absolute indications include progressive neurologic deficit (e.g., worsening motor weakness, bowel or bladder dysfunction attributable to compression), severe and progressive deformity with significant coronal or sagittal imbalance causing inability to stand or walk, and intractable pain accompanied by structural pathology that is unlikely to respond to conservative care.

Relative indications are more nuanced and often controversial. Many patients present with “intractable” pain, but pain is multifactorial and may not improve proportionally with deformity correction. Likewise, quality-of-life decline may arise from comorbidity, deconditioning, or psychosocial factors. In these gray zones, the decision to operate depends on the probability that surgery will meaningfully improve function, the magnitude of surgical risk, and the patient's values and tolerance for complications.

A helpful approach is to explicitly identify the primary surgical target: neural decompression for leg-dominant symptoms; stabilization for painful instability; alignment restoration for imbalance-driven disability; or a combination. When the target is clear, the surgical plan can be matched to the least invasive strategy likely to achieve durable benefit.

### 4.2 Preoperative assessment and risk stratification

#### 4.2.1 Patient factors

Age alone is an imperfect proxy for surgical risk; physiologic reserve, frailty, and comorbidities are more predictive (20). Preoperative evaluation should include bone health assessment (DEXA scanning when appropriate), nutritional status, anemia screening, diabetes control, smoking status, cardiopulmonary evaluation, and review of anticoagulation needs. Osteoporosis is particularly important because low bone mineral density increases the risk of screw loosening, proximal junctional failure, and pseudarthrosis.

Medication review and optimization can reduce perioperative complications. For instance, management of anticoagulants and antiplatelet agents requires coordination with prescribing clinicians to balance thrombotic and bleeding risks. Glycemic control and infection prevention protocols may reduce surgical site infection rates.

Psychological readiness and expectation management are central. Patients may seek surgery hoping for complete pain relief, rapid return to high-level activity, or cosmetic correction. Surgeons should discuss realistic outcomes, recovery timelines, activity restrictions, and the possibility of staged procedures. Informed consent must include a candid discussion of complications and revision risk, particularly for long constructs.

#### 4.2.2 Deformity factors

Deformity assessment includes curve magnitude, flexibility, sagittal balance, and the distribution of stenosis and instability (21). Flexibility can be assessed with bending radiographs or supine/prone imaging. Rigid deformities may require osteotomy to restore lordosis and balance, whereas flexible deformities may be addressed with interbody techniques and positioning.

Sagittal alignment is a key determinant of both disability and surgical complexity. Large SVA, high pelvic tilt, and substantial PI–LL mismatch are associated with worse baseline function and may necessitate lordosis restoration (13, 22). Classification systems such as the SRS– Schwab adult spinal deformity classification provide a structured way to describe deformity and have been linked to health-related quality of life (1).

Finally, the planned distal fixation (e.g., stopping at L5 versus extending to the sacrum or pelvis) influences both biomechanical demands and complication profiles. Pelvic fixation may improve construct durability in long fusions, especially when lumbosacral disc degeneration is present, but it increases surgical invasiveness and may affect postoperative mobility.

### 4.3 Selection and evolution of surgical strategies

#### 4.3.1 Decompression versus decompression with fusion versus osteotomy correction

Surgical strategy selection can be conceptualized along a spectrum.

**Decompression alone** may be appropriate for carefully selected patients with leg- dominant symptoms from stenosis, minimal or no instability, relatively small curves, and acceptable sagittal alignment. The main appeal is lower surgical morbidity. The main risk is postoperative progression of deformity or iatrogenic destabilization, which can lead to recurrent symptoms (9).

**Decompression with limited fusion** is often chosen when decompression is needed in the setting of instability (e.g., degenerative spondylolisthesis, significant lateral listhesis) or when a focal painful segment is identified. Limited fusion aims to balance symptom relief with acceptable risk, but adjacent segment degeneration and curve progression remain concerns.

**Long-segment fusion with deformity correction** is considered for patients with significant coronal and/or sagittal imbalance, progressive deformity, and substantial disability. These operations may include multilevel interbody fusion to restore disc height and lordosis, posterior column osteotomies, or more powerful three-column osteotomies for rigid kyphotic deformities. The potential benefit is meaningful improvement in global alignment and function. The cost is higher blood loss, longer operative time, greater physiologic stress, and increased complication and revision risk (23).

Selection should prioritize the patient's dominant disability driver. A patient with mild imbalance but severe unilateral foraminal stenosis at one level may not benefit from a long reconstruction. Conversely, a patient with profound forward stooping and inability to stand may not improve with decompression alone.

#### 4.3.2 Selection of fusion levels

Choosing the upper instrumented vertebra (UIV) and lower instrumented vertebra (LIV) is among the most consequential decisions in ADS surgery (24). Short-segment constructs may preserve motion and reduce invasiveness but may fail if deformity progresses above or below the fusion. Long constructs can improve alignment but increase the risk of proximal junctional kyphosis/failure, implant complications, and the physiologic burden of surgery (23, 25).

Stopping at L5 may preserve some lumbosacral motion, but it places high stress on the L5–S1 segment, which may already be degenerative. Extending fusion to S1 and/or the pelvis can improve distal stability and reduce the risk of distal junctional problems, particularly in long constructs, but it increases surgical magnitude and can affect sitting and gait mechanics. UIV selection often balances avoiding stopping at the thoracolumbar junction (a transition zone prone to junctional problems) against the desire to limit surgical extent. Patient- specific factors such as bone quality, thoracic kyphosis, pre-existing junctional degeneration, and sagittal profile influence this choice. Prophylactic strategies (e.g., junctional ligament augmentation, hook constructs, cement augmentation in osteoporotic bone) may be considered, though evidence continues to evolve (26).

#### 4.3.3 Role of minimally invasive surgery (MIS)

Minimally invasive approaches have expanded in adult deformity care, with goals of reducing blood loss, soft-tissue disruption, infection risk, and recovery time (27). Techniques include minimally invasive decompression, percutaneous instrumentation, and lateral or oblique lumbar interbody fusion (LLIF/OLIF) to restore disc height and achieve indirect decompression (28).

In selected ADS patients, MIS strategies can address segmental instability and foraminal stenosis while improving coronal alignment through cage placement and disc height restoration. Staged approaches (e.g., lateral interbody work followed by posterior percutaneous fixation) may permit meaningful correction with less physiologic stress.

However, MIS has limitations. Severe sagittal imbalance or rigid deformity may require osteotomy and open exposure. Indirect decompression may be insufficient when stenosis is severe, calcified, or associated with fixed facet hypertrophy. In addition, MIS techniques have learning curves and can still carry risks of neurologic injury, psoas-related symptoms, cage subsidence, and nonunion.

Therefore, MIS should be viewed as a tool within a broader armamentarium, selected based on deformity flexibility, alignment goals, neural compression pattern, and patient frailty (29).

#### 4.3.4 Importance of sagittal balance reconstruction

A central lesson from adult spinal deformity research is that restoring physiologic sagittal alignment is strongly associated with improved outcomes. Excessive positive sagittal balance correlates with pain and disability, and alignment targets such as SVA, PT, and PI–LL mismatch have been linked to patient-reported outcomes (6, 30).

In ADS, sagittal reconstruction often requires restoring lumbar lordosis, particularly in the lower lumbar spine where lordosis is normally concentrated (31). Interbody fusion can help regain disc height and segmental lordosis, while osteotomies may be needed for rigid kyphosis. Yet reconstruction must be individualized: overcorrection in older adults may increase junctional stress, while undercorrection may leave persistent imbalance and dissatisfaction. Contemporary alignment planning increasingly accounts for age-adjusted targets and the patient's compensatory reserve.

Ultimately, sagittal balance reconstruction is not a purely radiographic pursuit. The aim is to reduce compensatory energy expenditure, improve horizontal gaze and posture, and enhance walking and standing capacity.

#### 4.3.5 Two-stage surgery and dynamic stabilization as alternative or adjunctive strategies

In elderly or medically complex ADS patients, the morbidity of single-stage long-segment reconstruction can be prohibitive. Two-stage surgery has emerged as a risk-mitigation strategy, wherein anterior or lateral interbody procedures are performed in a first stage to restore disc height, achieve indirect decompression, and improve coronal alignment, followed by posterior instrumentation and fusion in a second stage after a period of physiological optimization (32). Recent studies have further explored this approach in osteoporotic or high-risk populations (33, 34). This approach may reduce operative time per stage, limit intraoperative blood loss, and allow interval recovery, thereby decreasing perioperative complication rates in frail patients. Although two-stage surgery extends total treatment duration and may increase overall resource utilization, it can expand surgical candidacy for patients otherwise considered high-risk for a single prolonged procedure (35).

Alongside fusion-based strategies, dynamic stabilization offers an alternative paradigm for select ADS patients. Devices such as pedicle screw-based dynamic rods, interspinous spacers, or ligament augmentation aim to provide segmental stability while preserving motion and reducing adjacent segment stress (36). In ADS, dynamic stabilization is most often considered for patients with mild to moderate deformity, symptomatic instability without severe sagittal imbalance, or as a supplement to decompression in cases where the risk of fusion-related complications is elevated due to poor bone quality or high comorbidity burden. While current evidence remains limited to relatively small case series, proponents suggest that dynamic stabilization may achieve acceptable pain relief and functional improvement with lower surgical invasiveness and preservation of future treatment options. However, its role in progressive deformity or sagittal imbalance is limited, and long-term durability compared to fusion remains uncertain.

Both two-stage approaches and dynamic stabilization illustrate a broader shift in ADS surgery toward risk-stratified, adaptive strategies that prioritize patient safety and functional preservation alongside deformity correction. Future comparative studies are needed to define patient selection criteria and long-term outcomes for these techniques relative to conventional single-stage long-segment fusion.

### 4.4 Complications and revision surgery

ADS surgery is associated with a broad complication spectrum. Understanding these risks is essential for counseling, prevention strategies, and realistic decision-making.

**Proximal junctional kyphosis (PJK) and proximal junctional failure (PJF)** represent a major source of reoperation after long constructs. Risk factors include older age, osteoporosis, large sagittal corrections, rigid constructs, and pre-existing thoracic kyphosis. PJF may involve vertebral fracture, implant pullout, or soft-tissue failure, and it can cause pain, neurologic deficit, or progressive deformity.

**Pseudarthrosis** (nonunion) can occur particularly at the lumbosacral junction and in long fusions (37, 38). It may present with persistent pain, rod fracture, or loss of correction. Risk factors include smoking, poor bone quality, malnutrition, and inadequate fixation. Strategies to reduce risk include optimizing bone health, using interbody support at high-stress levels, and considering biologic adjuncts when appropriate.

**Implant failure** includes screw loosening, rod fracture, and cage subsidence (39). These problems are more likely in osteoporotic bone, with large corrections, and at junctional areas. Rod fracture often reflects cyclic fatigue in the setting of incomplete fusion and may necessitate revision.

**Infection** risk increases with longer operative time, greater blood loss, diabetes, and obesity. Infection can lead to prolonged antibiotic therapy, debridement, and potential hardware removal.

**Neurologic complications** range from transient postoperative numbness to permanent deficit (40, 41). Risk is influenced by the extent of correction, osteotomy use, and the severity of preoperative stenosis. Intraoperative neuromonitoring is commonly used, but it does not eliminate risk.

**Medical complications** include cardiopulmonary events, thromboembolism, delirium, ileus, and urinary complications (42). These are particularly relevant in older and frail patients. Enhanced recovery pathways, multimodal analgesia, early mobilization, and careful perioperative medical co-management can reduce some risks.

Revision surgery is a significant concern because it amplifies cumulative risk and cost (43). Importantly, the possibility of revision should be considered during the first operation: robust distal fixation, thoughtful alignment planning, and junctional protection may reduce later failure. Nonetheless, even with optimal planning, some patients will require revision due to biologic limitations and the mechanical demands of long constructs.

These realities underscore the central tension in ADS surgery: while reconstructive operations can produce substantial functional improvement, they are high-risk interventions that must be matched carefully to patient goals, physiologic reserve, and the expected durability of benefit.

## 5 Conclusion and future directions

### 5.1 Summary of main conclusions

ADS is a prevalent and disabling condition driven by degenerative changes and compounded by global alignment imbalance and neural compression. Diagnosis is challenging because symptoms are non-specific and overlap with multiple degenerative and non-spinal disorders; accurate assessment requires careful history, examination, and standing full-spine radiographs with attention to spinopelvic parameters, complemented by CT and MRI.

Pain management must address mixed mechanical, neurogenic, and myofascial generators and should be delivered as an integrated, goal-oriented, and longitudinal strategy. Physical therapy and rehabilitation, rational pharmacologic therapy tailored to geriatric risk, and targeted interventional procedures each have roles, but none are universally sufficient alone. Surgical decision-making in ADS demands balancing potential functional gains against substantial complication and revision risks. A patient-centered approach clarifies the primary disability driver (stenosis, instability, imbalance), aligns surgical goals with the least invasive durable strategy, and emphasizes realistic expectations through shared decision-making (19). Multidisciplinary care involving spine surgeons, physiatrists, pain specialists, physical therapists, and medical co-management teams can optimize outcomes.

### 5.2 Future perspectives

Several directions are likely to improve ADS care.

#### 5.2.1 Precision medicine and prognostic modeling

Better prediction of curve progression, pain trajectories, and surgical benefit would enhance patient selection and counseling. Models that integrate radiographic parameters, bone health, frailty indices, and patient- reported outcomes may outperform single-variable heuristics (20). Emerging tools such as radiomics and machine learning may help characterize degeneration patterns and predict mechanical failure risk, but prospective validation and interpretability are essential before broad adoption.

#### 5.2.2 Technological innovation

Navigation, robotics, and augmented reality hold promise for improving instrumentation accuracy, reducing radiation exposure, and facilitating minimally invasive approaches, especially in complex deformity and dysmorphic anatomy (27). Advances in biomaterials, surface technologies, and biologic augmentation may enhance fusion rates, particularly in osteoporotic bone.

#### 5.2.3 Optimizing non-surgical therapies

High-quality trials are needed to define evidence- based rehabilitation protocols tailored to ADS phenotypes (imbalance-dominant vs. stenosis-dominant) and to evaluate combined pathways that integrate injections, behavioral strategies, and exercise. Given the chronic nature of ADS, sustainable home-based and digitally supported programs may be particularly valuable.

#### 5.2.4 Long-term outcome studies

Comparative effectiveness research over 10 years or more is required to understand the durability, quality-of-life impact, and cost-effectiveness of different strategies, including limited surgery versus long reconstruction, and MIS versus open techniques (43). Such data should include older and medically complex patients who are often underrepresented in trials, and it should incorporate outcomes meaningful to patients, such as independence, mobility, and caregiver burden (19).

In conclusion, ADS management is evolving toward individualized alignment-aware assessment and treatment, with a growing emphasis on function, safety, and shared decision- making. Continued integration of rigorous outcomes research, technological advances, and multidisciplinary care pathways offers the best prospect for improving the lives of patients with this challenging condition.
